# “It Was Me on a Good Day”: Exploring the Smart Drug Use Phenomenon in England

**DOI:** 10.3389/fpsyg.2016.00779

**Published:** 2016-05-27

**Authors:** Elisabeth J. Vargo, Andrea Petróczi

**Affiliations:** Pharmacy and Chemistry, School of Life Sciences, Kingston UniversitySurrey, UK

**Keywords:** cognitive enhancing drugs, grounded theory, normalization, drug instrumentalization, students

## Abstract

The non-medical use of prescription medication for the pursuit of increasing cognitive and intellectual capacities (defined neuroenhancement) has received growing attention from the scientific community and policymakers alike. To date, limited qualitative data exist exploring the nature of the phenomenon, especially as a potentially emerging trend among university students in England. Existing American literature suggests that students believe that neuroenhancement helps the individual to maximize his/her time, consenting a suitable balance between work and leisure. Students’ motivation to experiment with neuroenhancement appears to be more in line with a need to regulate emotions surrounding study/work settings than to actually improve cognitive abilities beyond normal levels. This study aimed to qualitatively explore representations, motivations, beliefs, and consumption styles of a cohort of university student users residing in England. Through snowball sampling, 13 informants were contacted and interviewed regarding their experience with neuroenhancers. Narrations were analyzed and interpreted using qualitative analysis software and Grounded Theory methodology. Participants belonged to a broad variety of university courses and were predominantly habitual consumers of modafinil. Neuroenhancers were acquired either through friends or via the Internet. Motivations regarded the need to “catch up” and be on par with high achieving students. The entire cohort had previously experimented with other psychotropic substances. Synthetic compounds in particular were believed to be “gateway” drugs to using neuroenhancers. Experimentation with neuroenhancement can be seen as a self-governing strategy aimed at achieving continued focused productivity. Participants acknowledged sustainable benefits in neuroenhancement as it optimized work performance. The majority of the cohort also contemplated the possibility of using these drugs in the future once they entered the workforce. Neuroenhancing drug users expressed “situated morality,” differentiating between using these substances for assessments (exams) or during revisions, finding only the former as an immoral conduct. In the present scenario, it appears that neuroenhancement is practiced by small numbers of students. Nonetheless, the instrumental views of psychotropic substances held by many young adults and the globalization of these practices make the normalization of neuroenhancement a plausible possibility of the future.

## Introduction

Humanity has attempted to increase cognitive ability since very early in history. In ancient Greece, rosemary twigs were placed in scholars’ hair with the hope to improve memory (Cakic, 2009). Traditional Chinese medicine has developed over thousands of years formulas reputed to improve cognitive abilities and concentration (Howes and Houghton, 2003). Today, the psychopharmaceuticalization of society (Goodman et al., 1996) has introduced prescription medications (modafinil, methylphenidate, phenethylamines, etc.) which can be used to increase cognitive performance in individuals suffering from mental health conditions such as narcolepsy, attention deficit disorders and shift work sleep disorder (Greely et al., 2008; Farah et al., 2014). Practices involving the non-medical use of such medications on the part of healthy individuals have been coined with the term pharmacological neuroenhancement, and have received growing attention on the part of the scientific community (Quednow, 2010; Repantis et al., 2010; Wolff and Brand, 2013).

In particular, an area of scientific interest resides in the use of these medications on the part of university students (Hall et al., 2005; Varga, 2012). Although it is still unclear if this trend is actually growing within the young adult population (Partridge et al., 2011; Ragan et al., 2013) or if it is a facet of a generalized aptitude on the part of western cultures to “medicalize” mental life (Coveney et al., 2012), ethical and policymaking issues still arise from this phenomenon. Besides whether neuroenhancement constitutes cheating (Lucke, 2012; Bell et al., 2013), it can also be hypothesized that these practices may become normalized, considering their appeal to younger adults entering the workforce (Wolff et al., 2014). If this population reputes these substances as instrumental to reach the full potential of their cognitive capacities, they may very well prolong neuroenhancement later in their life. Neuroenhancement already appears to be more or less prevalent in working populations (Maher, 2008; Banjo et al., 2010; Racine and Forlini, 2010; Wiegel et al., 2015). In a previous study, we found that socio-economic factors related to the competitiveness of the job market and preoccupations regarding occupational stability promoted a willingness to experiment with neuroenhancers (Vargo et al., 2014).

Neuroenhancement’s actual efficacy in improving intellectual performance in healthy populations is yet to be established (Farah et al., 2014) and has not been proven safe (Maier and Schaub, 2015). The addiction risk posed by these drugs is still in debate but nonetheless, concerning side effects such as psychosis, insomnia, and irritability may arise from the use of these substances (Hysek et al., 2014).

### Prevalence

According to survey data, significant portions (between 5 and 35%) of the North American student population utilize prescription medication to aid their cognitive abilities (Wilens et al., 2008). In the U. S., greater prevalence of use has been found among white male students, and members of fraternities and sororities (Hall et al., 2005; McCabe et al., 2005). Prevalence rates in Europe remain unclear, although they appear to be lower compared to North America (Maier and Schaub, 2015). In a study surveying UK students, less than 10% reported lifetime prevalence, but one third expressed an interest in experimenting with neuroenhancement (Singh et al., 2014). According to authors, low prevalence and high interest among British students could be moderated by the scarce availability of neuroenhancers (Singh et al., 2014).

It has also been debated that in the U. S., the endemic prescription of Ritalin to minors in the 1990s has contributed to the widespread use of prescription stimulants among young adults in higher education (Conrad and Potter, 2000; Loe, 2008). Although this interpretation is coherent within the North American context, it does not justify the apparent growing popularity of these practices in European contexts. Prescriptions to young children of these medications are less common in the UK, and have only risen in the last decade (Southall, 2007).

### User Characteristics and Motives

Individuals who use neuroenhancers appear to have lower levels of self-efficacy and score higher on neuroticism scales (Maier and Schaub, 2015). They are also more likely to abuse other legal and illegal substances for self-medication (Novak et al., 2007; Singh et al., 2014). According to Quintero et al. (2006), the unprescribed use of medications for physical, social and psychological needs on the part of healthy young adults is part of broader normalization processes which involve the medicalization of a variety of states of being. College students view prescription drugs as a safer and more socially acceptable alternative to using “harder” drugs (Quintero et al., 2006; DeSantis and Hane, 2010). In this prospect, the self-medication hypothesis has been proposed by several authors as an explanation to these contemporary trends. Ford and Schroeder (2008) have found that students reporting higher levels of depression were more likely to experiment with prescription stimulants. Wolff and Brand (2013) found that university students view neuroenhancement as an acceptable means to cope with stress related to scholastic demands.

Qualitative research exploring neuroenhancement has evidenced that this practice is embedded in a multifaceted life characterized by high demands (Hildt et al., 2014). Students believe that neuroenhancement helps the individual maximize his/her time, thus consenting a suitable balance between work and leisure (Hildt et al., 2014). Moreover, Vrecko (2013) argues that students’ motivation to experiment with neuroenhancement is more in line with the need to regulate emotions (increase enjoyment, interestedness, and drivenness) than to its actual capacity of increasing cognitive performance. According to de Souza (2015), biomedical discourses characteristic of contemporary society dominate students’ beliefs in regards to the efficacy of neuroenhancement. The body-as-machine metaphor is used to interpret the problems of college life, and pharmaceutical drugs are viewed as a quick fix to the “mechanical” problems of lack of time, motivation and stress (de Souza, 2015). This cultural representation is amplified when exploring the views of students who have obtained prescriptions for neuroenhancers: the boundaries between treatment and enhancement appear blurred, and prescription is used as a form of legitimization (Petersen et al., 2015a).

### Aims and Objectives

Aims and objectives of the present study regarded the investigation of motivations, beliefs and attitudes tied to neuroenhancement on the part of university students, using qualitative and ethnographic techniques that give value to the individuals’ subjective experience. England is characterized by different sociocultural factors than the North American context, thus it is relevant to provide a qualitative account of the neuroenhancement phenomenon within this context. In the UK, Ritalin (methylphenidate) is a Class B drug while modafinil is unclassified and more easily purchasable via the Internet (Advisory Council on the Misuse of Drugs [ACMD], 2015). Moreover, it is to be noted that the students participating in this study belong to the first wave of students who are paying full tuition fees as of September 2012 (£9,000 a year), which amount for increased pressure to do well in their studies to secure a good job upon graduation. It is to be noted that the present study does not aim to provide data representative of the entire population of neuroenhancement users, or of the subpopulation of University students in England using these types of enhancers. Moreover, our intent revolves around the need to explore psychosocial variables involving networks of users, outlining their specificities and identifying those elements attributable to a more generalized drug using culture.

### Research Sample

For the purpose of this study, 13 informants were approached through ethnographic methodology, using snow-ball sampling (Fountain, 2000). The inclusion criterion was having used a neuroenhancer at least once without a medical prescription. Once key figures were contacted, these individuals were asked to help find more participants who belonged to the same social network of users.

**FIGURE 1:**
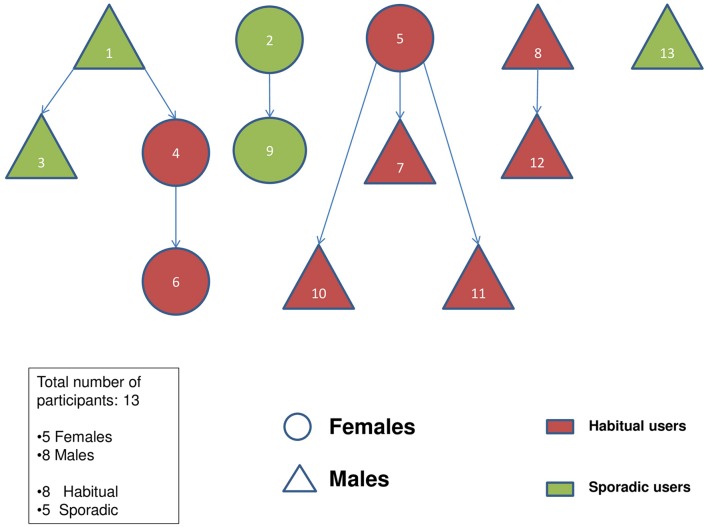
**Sociogram of the study population.** Numbers represent the order of recruitment.

Participants belonged to a small age range varying between 21 and 24 years old (*M* = 22.5 ± 0.9 years). The sample contained more males (*n* = 8) than females (*n* = 5), reflecting survey results which evidence a propensity on the part of male students to experiment with these drugs (Hall et al., 2005; Vargo et al., 2014). The entire sample possessed a bachelor’s degree and three participants were pursuing a postgraduate degree. Subject studied during undergraduate studies varied greatly, with four participants in the social sciences, four in computer and engineering and five in biology and medicine. Again reflecting survey results (McCabe et al., 2005), nine participants were white British, two were white Canadian and two were Pakistani. Within the sample, five participants had experimented with neuroenhancing drugs less than 10 times (“sporadic” users) while eight had used them habitually for a limited or extended period of time (“habitual” users). **Figure 1** depicts the sociogram of the study sample and the snow-ball sampling process.

## Materials and Methods

Qualitative research plays a fundamental role in the comprehension of the psychotropic drug use phenomenon (Fountain, 2000). Ethnographic research can provide qualitative information that not only contributes to a clearer understanding of new drug trends, but can also provide a term of comparison for quantitative research designs.

The methodologies utilized in this study are characterized by flexible data collection and an unstructured initial hypothesis. GT methodology and the Life-Story interview technique (Strauss and Corbin, 1990; Atkinson, 1998) permit the same themes proposed by the participants to become object of interpretation, maintaining the original linguistic code adopted by the sample. Through the Life-Story interview, participants are free to tell their own “story” in regards to neuroenhancement use. Subsequently, associations between thematic categories emerging from GT methodology determine the creation of a “single” storyline (Strauss and Corbin, 1990) which narrates the sample’s experience and relationship with neuroenhancement. Coherently to a constructionist approach which interprets reality as resulting from a shared social construction (Kelly, 2003), it was chosen to interpret the research samples’ narrations as “sense-making” of the investigated phenomenon. Considering the debates on the morality of neuroenhancement (Goodman, 2010) and that the non-medical use of some neuroenhancers (e.g., Ritalin) is illegal, it was acknowledged that participants would show resistance upon the request to share their personal experiences. Using a non-judgemental attitude and a tolerant approach, it was possible to overcome the suspicion and resistance that people would usually enact when prompted regarding their illicit conducts (Lambert, 1990).

### Procedure

Participant recruitment followed the snowball sampling method. This technique assumes that the members of a social network are able to identify better than the researcher potential participants and are better informed in regards to the practices the researcher wishes to investigate (Lambert, 1990). Neuroenhancing substance users (participants 1, 2, 5, 8, and 13 in **Figure 1**) were initially identified (from previous studies and the researcher’s social network) as key informants for the subsequent snow-ball sampling process. During data collection, the researcher took notes of the snow-balling process to aid the interpretation of results.

In agreement with the key informants, potential participants were contacted directly by the researcher and details regarding the study’s objectives and procedure were first handedly described to them. Once the potential participant agreed to participate in the study, a meeting was arranged to carry out the interview. The setting of the interviews was a quiet and private area, where the interviewee could feel comfortable and at ease. As suggested by Atkinson (1998), participants were told before the actual interview to think about and try to recall significant events of the past and present which they found relevant to their experience with neuroenhancement. This would help the sense-making process and avoid that the interviewee presents a mere list of events. The aim of this type of interview is in fact to achieve an actual *story* of the individual’s experience, with characters, setting, plot, conflict, and resolution (Atkinson, 1998).

Interviews lasted approximately 45 min and were divided into three parts, each lasting about 15 min. According to Atkinson (1998), separating the phases of the interview helps the participant reflect and elaborate the contents he/she wishes to share. Each subpart was introduced by a prompt enquiring about the interviewee’s relationship with neuroenhancement in the past, the present, and the future.

Interviews were carried out between March 2014 and March 2015. These were audio recorded and subsequently transcribed, omitting parts where the interviewee made reference to private information that could endanger their anonymity (names, places, etc.). The study was approved by the Kingston University Faculty of Science, Engineering and Computing Research Ethics Committee. At completion of the interview, participants were rewarded for their time and contribution with a £20 gift voucher.

### The Life-Story Interview

The Life-Story interview (Atkinson, 1998), a discoursive and non-directed interview based on the active participation of the research sample in the creation of an interpretation, was chosen as our primary instrument for data collection. This interviewing technique views the narration of the *story* as the creation of a shared truth between the narrator and the listener. The story represents a privileged form of a unique personal expression, thus a way to access the cognitive world and representations of the storyteller.

The Life-Story interview is relatively unstructured and based on cooperation. The interviewer abstains from commenting or providing opinions in regards to the participant’s conduct, and allows the interviewee to choose which topics and subjective experiences he/she wishes to share in relation to the interview’s queries. In the case of the present study, three prompts were used to develop the interview:

(1) To describe the first time they experimented with neuroenhancement, their impressions, their beliefs before and after this event. To describe how the substance affected their body and their performance.
(2) The second prompt regarded the interviewee’s relationship with neuroenhancers in the present: how did their use evolve from the first time they experimented with them and how the effect changed or maintained itself. The interviewee was then asked what opinions and attitudes their friends and family had in regards to neuroenhancement. In the case the interviewee was unaware of their friends’ and family’s attitudes, he/she was asked to imagine how they would react if they found out about their conduct.
(3) The third prompt asked the participant if he/she intended to use neuroenhancers in the future and under which circumstances. The interviewee was asked to imagine this hypothetical scenario, as well as what kind of motivations would lead to this decision. The participant was then asked if he/she had ever used other psychotropic substances and how they were similar or different to the neuroenhancers they had experimented with.

These prompts aimed at collecting data regarding participants’ attitudes, beliefs, and consumption trajectories in relation to neuroenhancement. In particular, the first question aimed at comparing beliefs regarding neuroenhancers before and after drug experimentation, and motivations tied to the intiation of this conduct. The second question aimed at collecting information regarding consumption trajectories, as well as participants’ beliefs regarding ingroup and outgroups’ attitudes and representations of psychotropic drug use. The third question explored intentionality of using neuroenhancement in the future and psychotropic drug use in general.

The life-story interview is a qualitative research method which does not aim at confirming the presence or absence of specific categories, but intends to collect an uncountable number of models and meanings that permit the formulation of inductive hypotheses. According to Atkinson (1998), historical truth is not the main issue when assessing a story’s reliability. The possibility of considering the story worthy of trust is more relevant to the research process. The objective is not to measure an objective truth, but to collect information regarding the subjective experiences of a social event.

### Grounded Theory Methodology

The goal of GT methodology is to systematically explore the meanings that the study participants attribute to the social reality they belong to, in order to produce “plausible interpretations” of a process or an interaction (Creswell, 2008). The main approach consists in a constant comparison between the different phases of interpretation, following a circular process (Strauss and Corbin, 1998). The starting point is a “cognitive query” regarding a specific issue, in our case the meanings attributed to neuroenhancement.

Through the *coding* process, the narrations are fragmented and reassembled via an abstraction process. Initially, concepts are organized in codes that are as close-fitting as possible to the text and progressively, the categorization process promotes the abstraction of these concepts. A code therefore, describes a portion of the text (quotation) through a label representing the narrative theme. Following the GT method, units are chosen according to their *groundedness* (prevalence in the narrations) or to their significance for the researcher’s theoretical elaboration (Strauss and Corbin, 1990). The coding process is divided into three phases: open, axial, and selective coding. These do not follow a linear sequence but a circular one, as data and categories are constantly compared with each other in this process.

### Data Analysis

**FIGURE 2:**
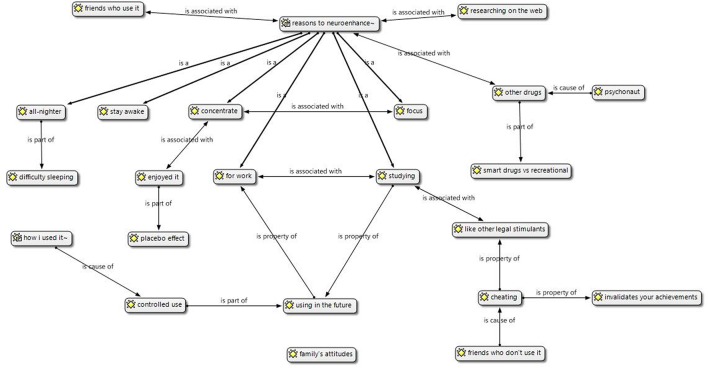
**Conceptual map depicting the sample’s storyline.** Nodes represent the most relevant codes according to groundedness and centrality in the interpretative process. Links represent relationships between codes.

This study utilized Atlas.ti software, which is tailor-made for GT methodology; and supports and organizes the coding procedure. Analysis of the relationships between codes is possible through the *query tool*, which analyses Boolean, logical, semantic, and proximal links between the categories. Atlas.ti also aids the analysis of relationships between conceptual categories and socio-demographic or other structural variables (i.e., gender, social network affiliation, drug used, and consumption style). Through this software program it is also possible to visually organize the codes emerging from the analytical process. **Figure 2** shows the conceptual map or *storyline* representing the organization of the codes. This organization is dictated by the GT methodology previously described, thus by the inductive–deductive process which aligns the researcher’s interpretation to the co-occurrence and groundedness of the narrations’ main themes.

## Results

The narrations of our study sample presented fairly coherent stories in regards to motivations, representations and effects experienced. As can be observed in **Figure 2**, reasons behind using neuroenhancers are semantically very similar (e.g., concentrate, stay awake, and focus). Regarding the type of neuroenhancers used, eight participants had tried modafinil (Modalert, Modavigil; a wakefulness promoting agent), two had also tried either Ritalin or Adderall, and three participants experimented with Adderall. Access to the prescription medications Ritalin and Adderall was determined by the fact that participants were in North America when experimentation with these substances occured, or someone who had these prescribed in North America provided them the drugs. According to the sample, prescription medications Ritalin and Adderall were very difficult to obtain in the UK whereas in North America, the use of these medications was very common among university students. No significant differences were found in regards to the effects provoked by these different compounds, thus the perceived effects were grouped for all three compounds.

The codification process resulted in the identification of 1593 quotations organized in 77 codes. The sample’s storytelling was centered around explaining and justifying the reasons behind their prescription stimulants use to improve academic performance. The *storyline* elaborated from interpretation was organized according to this recurring and preponderant theme.

### Motivations Leading to Neuroenhancement

The analysis of the sample’s narrations evidenced that an important motivation leading to experimentation with neuroenhancers was work management and the possibility to intensify working sessions within limited periods of time. Primarily, participants hoped neuroenhancement would help them to “pull an all-nighter,” boost their concentration, energy and motivation toward the task at hand. The need to resort to modafinil or other neuroenhancers derived from pressing deadlines or preoccupations with performing well.

“*There are people who I was aware of, who were just working way harder than me and weren’t taking any drugs at all. Maybe you’re just bringing yourself up to their level by making the use of chemicals.”* Brian, aged 22.

As a matter of fact, many participants although aware of the existence of these compounds, chose to use them during the final year of their degree, when the pressure to outperform increased. These participants would also actively purchase modafinil from the Internet, driven by their need of assistance in their work management.

Expectations relative to neuroenhancers were mainly formed from the experiences of peers’ already using these drugs or from the media. In general, participants would use Internet sites (e.g., Wikipedia, Reddit) to find more information on the effects of neuroenhancers and explore the experiences of other users. Moreover, these students were not preoccupied with negative outcomes and detrimental side effects: they viewed these substances as medications and had rarely heard of negative experiences on the part of their peers. Many students described not having particular expectations in regards to cognitive enhancement and described their motivation to try as determined by situational needs.

*“I didn’t really think much of it, I just took it to see if it would really work.”* Caroline, aged 21.

Moreover, a small number of participants were motivated to try neuronhancement out of curiosity, as they belonged to social networks of individuals who were using these drugs. Reassured by their peers’ positive experiences and motivated by the growing popularity of “smart drugs,” these participants usually experimented sporadically and never acquired the substance directly.

*“To be honest, the first time I tried it, it was more because I was bored of revising and writing the essays, rather than the fact that I necessarily needed to focus more. I thought ‘maybe it will be easier, maybe it’ll be more interesting, and I’ll see what it’s like’. I got it because he was getting some anyway, so he just got me some to try.”* Brenda, aged 24.

Before experimenting with neuroenhancement, the entire sample had tried at least one illicit substance. The most commonly mentioned drug was cannabis, then MD/MA (or ecstasy). Other illicit substances mentioned were cocaine, speed (or base), MDA, hallucinogenic mushrooms, LSD, ketamine, 2C-B, 2C-I, 2C-E, and 25I. For some, MD/MA was considered a “gateway” drug to neuroenhancement: their initial experience with this drug led them to change their views in regards to synthetic compounds. After experimenting with them, participants saw synthetic drugs as less dangerous, thus contemplated the possibility of using them for purposes different from entertainment. Moreover, other participants considered themselves “drug effect explorers” or, as defined by the drug using community, *psychonauts*: this term defines individuals who see psychotropic substances as a means to explore new experiences and new ways of relating to their environment. Drugs can be used rationally to enhance one’s life experiences and are considered privileged keys to access different levels of consciousness.

### Experimentation with Neuroenhancement

The majority of participants had a positive experience with neuroenhancing drugs. Actual effects met their expectations and the substance assisted them in meeting their goals. In line with their expectations, participants felt more awake and focused, “*on the ball*” and more interested in their work. Interestingly, many acknowledged the possibility of experiencing a “placebo effect.” Although it was recognized that neuroenhancers did not actually change the way participants thought or that they actually “created” motivation, effectively concentrating and focusing on their tasks provided them with a sense of enjoyment. In this sense, it can be stated that neuroenhancement provides an experience that in general, is rewarding for the user both at a cognitive as at an affective level. As would be expected, participants who described the experience as pleasurable, tended to intensify and habitually use neuroenhancers.

*“It was me on a good day, it wasn’t any better than me on a good day, it was me on the best day I could ever have.”* Amber, aged 22.

“*I don’t even know why, because I know I could do without them, it kind of makes me feel more confident. If they’re there, I know I could have a good day of work if I needed to but I could do without it.*” Mohammed, aged 22.

In general, participants described a primary effect that would last 5–8 h, and a feeling of being awake that would prolong itself for several more hours. The outcomes of neuroenhancement were reputed similar to those felt when drinking coffee, although participants recognized distinct differences in the capacity to remain focused and concentrated. Effects depended on the dosage taken, and many participants experimented with different amounts to achieve the desired effect. Taking half a pill for example, provided alertness while taking a full pill would provide more noticeable effects, both physically and psychologically. In particular, a full pill would give a “buzz,” a heart rush and a significant change in their motivational drive. Some participants described the “rush” as similar to that experienced from MD/MA or cocaine but to a lighter extent.

Consumption patterns varied widely, with some participants taking just a quarter of a pill sporadically to others who would take several pills during a single session. Intense use, such as using the substance for several days in a row, was described as extremely tiring for the body, and some believed that this conduct led to habituation. The majority of participants would take a half or full pill in the morning to take advantage of the effect during the day. Some would take a pill in the evening to work throughout the night. This modality usually resulted in a negative experience, as it affected sleeping patterns. Moreover, the majority of participants would use neuroenhancing substances for study revisions or to complete coursework (writing essays, project assignments). Often other stimulants were used simultaneously, such as coffee or caffeine pills, and energy drinks. Some, in particular males, had experimented with these substances in different contexts than solitary studying, and had neuroenhanced at work, for job interviews, during work out sessions, when clubbing or during examinations.

“*And then I also took half before I went for an exam. To be honest that was one of the best effects I had out of it… I think it definitely improved my memory in that case.*” Toby, aged 22.

In general, participants did not complain of particular side effects although the most frequently mentioned was insomnia. This side effect mainly concerned those using modafinil. When experiencing unwelcomed side-effects, participants adjusted their consumption style in order to avoid this side effect, or utilized other psychotropic substances (cannabis) to relieve their difficulty sleeping. A minority of the sample complained that using the drug when living a state of stressfulness would worsen the feeling and provoke panic and excessive worrying. Again, participants reported that they adjusted their consumption pattern in order to control this side effect.

Distractions from work would also provoke a state of uneasiness and distress. Some participants described being annoyed by distractions and avoiding social interactions. This feeling though depended on the intentionality of the user, as participants who were using neuroenhancers in work contexts contrarily described enjoying social interactions as they improved their communication style and eloquence. Participants described having a “craving” or “fixating” on doing something when under the effect of neuroenhancers, and for this reason keeping focused on the task at hand was important. This feeling would manifest itself in chain smoking, reordering objects or intense “Internet surfing.” Regarding physical side effects, some participants described having heart palpitations at the initial stages, loss of appetite and increase in thirst.

A duality emerged from the narrations in regards to the addictive quality of neuroenhancers. On one side, participants were informed that these substances were not physically addictive, and this was seen as a reassuring characteristic. On the other, some participants were preoccupied with psychological dependence, as they considered the possibility after continued use, of having to rely on these substances to carry out their work in an effective manner.

“*Thinking about what I’ve said it sounds like I’m addicted to them or something. The language that’s being used, I want to take it once a week, adding it to your daily life sounds quite similar to someone addicted to a specific drug, like coke, I’d only take it during the week ends. That’s how it starts, and then you’re taking it all the time.*” Paul, aged 24.

Participants viewed neuroenhancement as very different from using other psychotropic substances. The effects of illicit substances were seen as more intense and involving the whole body. Neuroenhancement on the other hand was perceived as more “psychological,” and not as physiologically overwhelming. Neuroenhancing drugs did not manifest any noticeable physical effects, whereas other drugs were much more visible. Many participants underlined the fact that neuroenhancers did not provoke particular negative effects once they wore off, contrarily to other drugs such as cocaine or MD/MA which are characterized by a “come down.” Moreover, neuroenhancement was seen as significantly different from other illicit drugs as the former were used for functional reasons and not recreationally.

### Attitudes of Participants’ Social Networks

Participants described belonging to social networks where the majority of peers did not use neuroenhancers. On the other hand, the three participants who had also experimented with neuroenhancement in North America described a different scenario where these substances were widely used. The cohort believed that non-users were better organized, more focused and therefore did not need to use neuroenhancers for their studies. According to participants, people who did not use neuroenhancers made this decision based on moral grounds, as they were against using drugs in general. Many participants shared that some of their peers and friends were curious about their experience with these substances. They believed that given the opportunity, more students would experiment with neuroenhancers.

“*Friends opinions usually start off negative just because they say you don’t know what it is, you go online. Until you don’t explain to them or you show them, then they’re like ‘Oh my god, I want to try some.’ When they learn about it, their opinion seems to change.*” Claire, aged 21.

The sample expressed concerns in regards to general attitudes which believed neuroenhancement was a form of cheating. Participants were worried that their choice might invalidate their achievements or that they would be kicked out of university or fired if they were exposed. In particular, habitual users discussed more of this topic, yet believed that this moral approach was inappropriate. Neuroenhancement was said to be the same as drinking coffee during studying sessions and did not change an individual’s capabilities. Some believed that using neuroenhancers during exams would be a form of cheating; using them during revisions was not unethical.

“*It’s kind of giving you an unfair advantage but then at the same time there are people who I was aware of, who were just working way harder than me and weren’t taking any drugs at all.*” John, aged 23.

Participants stressed that in comparison to individuals who were more capable at focusing and managing their commitments, they were not gaining an unfair advantage but were contrarily “catching up.” When considering peers who were as stressed or as under pressure as them, participants saw that neuroenhancement was providing them an unfair advantage. Nonetheless, the fact that these substances were widely known and easily purchasable on the Internet provided a justification to their conduct.

With very few exceptions who felt that their caregivers completely trusted their judgment and decisions, the sample believed that family members would disapprove of their experimentation with neuroenhancers. In general, family members were believed to have negative attitudes toward drugs, and neuroenhancers would be seen as belonging to the same category. The sample believed their family saw drugs as dangerous and taking drugs to study would be perceived as “crazy.” They would usually not discuss these experiences with their parents or siblings, as they were preoccupied with raising concerns.

### Intentionality to Use Neuroenhancement in the Future

The study sample believed that overall, neuroenhancement had a positive impact on their work commitments. In particular, it helped them stay awake and complete their work in restrained periods of time. Nonetheless, they believed that it did not “add” anything to their actual capabilities, and some were convinced that having better management skills would have provided the same contribution as neuroenhancement.

“*If I was getting behind work and I felt like I needed to catch up, if I felt I needed to get a lot of work done in a short period of time, there was a deadline moving, or if people around me start taking them I might feel maybe I should take them as well*.” Peter, aged 22.

With the exception of two participants, the whole cohort contemplated the possibility of using neuroenhancers in the future. The reasons behind this intentionality were the effectiveness of these substances and their affordable price. The possibility of using neuroenhancers was considered situation dependent, as participants believed that neuroenhancement was useful in times of intense stress and responsibilities. Some participants still possessed a “stash” of these drugs in case of need. As can be seen in **Figure 2**, participants viewed the possibility of neuroenhancing again as associated to their postgraduate studies or to a future job characterized by tight deadlines and individual projects. The possibility of being fired or getting in trouble in a work environment due to this practice was considered a deterrent from further experimentation.

“*There’s a big difference between taking it at university and taking it at work because work is the rest of your life, and having to take drugs to get through the rest of your life sounds terrible*.” Brian, aged 23.

Moreover, the fear of “losing control” over one’s drug using was considered another deterrent to further experimentation. Participants also contemplated the possibility of using other neuroenhancing drugs. According to their narrations, many other neuroenhancing drugs are available via the Internet and web forums provide valuable information on their efficacy. An example of alternative neuroenhancing methods is “stacking,” implying the use of cocktails of substances to reach optimal levels of alertness and concentration.

## Discussion

The study sample’s motivations to neuroenhance resided in their need to “catch up” and cope with their work related demands, in line with previous qualitative literature on the phenomenon (Repantis et al., 2010; Coveney, 2011; Vrecko, 2013). These findings also appear to be aligned with quantitative studies showing a propensity on the part of lower achieving students to use neuroenhancement (Benson et al., 2015).

Neuroenhancement was usually a solitary practice integrated with the way participants preferred to study. Modafinil was the most widely used neuroenhancer, as it was easily purchasable via the Internet and posed no legal consequences. In line with Singh et al.’s (2014) results, prescription medications Ritalin and Adderall appeared to be more difficult to obtain within the UK. The study’s participants who had tried these medications accessed them through individuals in their social network that had prescriptions (which were usually obtained in North America).

The decision to experiment with neuroenhancing drugs was also determined by their apparent growing popularity and by the attention they receive on Internet forums and the media. Participants believed these substances were safe, being medications. Moreover, their peers’ experiences with these drugs were generally positive, leading to a willingness on the part of some participants to try these substances without any specific need to “enhance.” In our sample, willingness to purchase modafinil via the Internet was associated with habitual use and intentionality to use neuroenhancers in the future.

Previous experiences with various psychotropic drugs led to a propensity to further experiment with neuroenhancement, confirming findings from previous literature (Novak et al., 2007). In particular, participants had broadly experimented with several synthetic substances which they conceived as a “gateway” to using other synthetic compounds for work related purposes. This representation confirms the idea that psychotropic drugs are integrated in western cultures, and seen as instrumental for the adaptation to modern life (Müller and Schumann, 2011). Moreover, the sample often practiced polysubstance use by simultaneously ingesting neuroenhancers with other legal stimulants to heighten their effect. Other psychotropic drugs such as cannabis were used by some participants to relieve the side effects caused by modafinil use. The instrumental view of psychoactive substances is further evidenced by the “psychonautical” culture (where drug experimentation is part of existential investigation) emerging from the sample’s narrations. This theme originating from the psychedelic subcultures of the 1960’s now appears to be a cultural value characterizing contemporary youth cultures (Schifano et al., 2003; O’Brien et al., 2015).

The sample’s experience with neuroenhancement led them to believe that these substances couldn’t actually change their cognitive and intellectual capacities, yet the majority continued their use and found them to be useful for their work performance. Although potential users believe prescription stimulants can improve cognitive abilities, research has shown little evidence of significant improvement in healthy populations. On the contrary, it is suggested that neuroenhancers may have a greater impact on mood and perceived motivation (Ilieva and Farah, 2013). Differently from legal stimulants such as coffee or energy drinks, neuroenhancers not only promoted wakefulness but the possibility “*to not worry about anything except for the task at hand.*” According to the narrations, their efficacy resided in helping them achieve a sense of focused productivity which fulfilled their motivational goals, consequently providing for the majority a sense of enjoyment. In this sense, neuroenhancers are not strictly viewed as a means to push the boundaries of what is possible for the individual but as a way to normalize performance during abnormal circumstances (Coveney, 2011).

Participants were aware of side effects consequential to neuroenhancement use and abuse. They demonstrated to be “rational” drug users by adopting strategies to control their use patterns and regulate consumption (Zinberg, 1984). These varied from reducing the amount of substance ingested to adjusting their times of consumption in order to avoid insomnia, which was the most frequent side effect mentioned by the sample. Moreover, the majority of participants viewed the use of neuroenhancement as circumstantial to specific moments of their existence (i.e., during periods of intense stress). Nonetheless, a dichotomy emerged from the narrations regarding the addictiveness of neuroenhancers. On one hand, these were not considered to be addictive, and this constituted a reassurance regarding their safety. On the other, some participants had experience of peers’ abuse and reliance on these substances, thus acknowledged the possibility of becoming “psychologically” addicted. Prevention and harm reduction strategies should address this ambiguity and better inform public knowledge regarding the meaning and psychological harm of addiction in its various forms (Ross et al., 2010). Moreover, research has demonstrated that students who neuroenhance adopt at-risk conducts which could lead to addiction (Hildt et al., 2015).

Another concern arising from the sample’s narrations regarded the morality of their conduct. Participants did not entirely believe that neuroenhancement constituted cheating, especially when carried out for revision or coursework completion. Using neuroenhancement for an exam or job interview on the other hand was reputed cheating. It appears that this conduct is practiced following a contextualized or situated morality. Similar to previous findings (Vargo et al., 2014), zero-sum situations elicit moral disagreement regardless of enhancement utilized. The need to enhance is a response to contextual demands linked to ecological pressures, evidencing its functional role in the daily routines of users. Attitudes of the general population toward neuroenhancement were perceived as negative. Participants believed that if their conduct were exposed, they would be fired or their achievements would be invalidated. Due to the fear of society’s negative judgement, participants held conflicting norms in relation to using medications for competitive needs, similarly to what has been found in athlete populations in relation to doping (Bloodworth and McNamee, 2010). Considering what has been learned from drug prohibition and anti-doping (Kayser and Smith, 2008), repression inevitably leads to a submersion of the phenomenon and consequently to increased difficulties when public health would aim at addressing the issue. Under a harm reductionist perspective, it would be important to not address this phenomenon using moralistic and purely bioethical paradigms (Ketchum, 2013), as these approaches produce social deviance and further harms, especially when neuroenhancing compounds are used for self-medication (Quintero and Nichter, 2011; Levinson and McKinney, 2013).

Beliefs regarding the effectiveness of neuroenhancing drugs led the majority of participants to imagine using these substances in the future. In this study, attitudes of non-users toward neuroenhancement appear to be negative. If the functionality of these drugs for users’ lives and goals emerge in other quantitative and qualitative studies in different contexts, a normalization of this conduct in future years can be hypothesized (Benanti, 2010). Our cohort resided in England but those who had also experienced with these drugs in North America described distinct differences in regards to the availability and popularity of neuroenhancement drugs. When also considering the notable attention these practices receive from the media (Partridge et al., 2011), a process of cultural accommodation and globalization of these practices is possible in the years to come.

Neuroenhancement seems to be an adaptation to work-hard play-hard lifestyles, as well as to the competitiveness of contemporary higher education. Borrowing from Foucault’s (1985) concepts relative to the *Technologies of the Self*, drug use conducts have an adaptive and functional role within the environmental setting in which they are carried out. Prescription stimulants can be seen as a strategy to govern the self, not just in relation to quantifiable results, such as grades or amount of work done, but also in relation to the affective experience of working hard and feeling on par with high achievers. Considering the evidence available and the absence of studies outside controlled laboratory experiments objectively verifying the efficacy of cognitive enhancers for healthy populations, it is difficult to confidently state in a quantifiable and objective manner that users are or are not actually enhancing their cognitive performance. Participants’ narrations nonetheless speak of advantages in terms of fulfilled accomplishments and focused productivity, and not in terms of quantifiable differences in their learning abilities. Although neuroenhancement drug users hold representations of cognitive enhancers as drugs capable of enhancing cognitive and intellectual abilities, they appear to be motivated to use these substances to keep up with academic demands, and not to push the limits of their abilities (i.e., being smarter or knowing more). Thus, their use is tied to the need to comply with and readjust their work performance to meet the day-to-day demands of their academic courses. Neuroenhancement can be seen as fulfilling efficacies both at a social as at a cultural level (Petersen et al., 2015b).

Outlining an articulate description of the phenomenon which considers the complexity of social attitudes, motivations, beliefs, and consumption styles seems coherent with the real-world applications of the findings of this study. Nonetheless, limitations reside in the small sample size and in the absence of quantitative information regarding personality and intra-individual variables. A limitation that can be identified in the methodology used in this study regards the risk of not fully satisfying theoretical saturation through the recruitment process. The snowball sampling process was in fact interrupted as participants were not able to identify more users and it was not possible to expand the study’s cohort. However, in qualitative research sufficiency of sample size is measured by depth of data rather than frequencies (O’Reilly and Parker, 2012). The high coherence identified in the participants’ narrations reassures that the data are reliable and relevant to understanding academic neuroenhancement in the English context. It can be hypothesized that the hidden population of neuroenhancing drug users is very contained in the geographic area assessed, and this may have been reflected in the snow-ball sampling process.

Moreover, a re-analysis of the narrations could involve more than one researcher in the coding process. Further investigation of this phenomenon could explore and compare the representations of young adults using neuroenhancement in work related contexts, or in different geographical settings. Future research should also investigate the neuro-biological effects neuroenhancing compounds produce on healthy individuals (Ilieva and Farah, 2013) as well as how perceived effectiveness and intrinsic motivation influence initiation and patterns of use (Ilieva and Farah, 2015).

## Conclusion

Our intent was to provide theoretical hypotheses that could contribute to the understanding of the phenomenon and promote effective preventive strategies. The sample used neuroenhancing substances to satisfy adaptive needs related to their work and academic demands. Substances were acquired from unsafe sources and many participants showed a willingness to resort to these compounds if they encountered stressful work situations in the future. These aspects should be taken into consideration in future harm reduction interventions. Understanding how an individual belonging to a specific social category constructs the usefulness of a psychotropic substance, and comprehending which motivations and beliefs lead him to experiment with it, are vital for the elaboration of appropriate and effective harm prevention.

## Author Contributions

EV designed the study and wrote the protocol with AP. EV performed the literature search, developed the interview protocol, carried out recruitment, collected the data, transcribed the interviews, analyzed the interviews, and drafted the first version of the manuscript. AP contributed to writing the manuscript. All authors contributed to and have approved the final manuscript.

## Conflict of Interest Statement

The authors declare that the research was conducted in the absence of any commercial or financial relationships that could be construed as a potential conflict of interest.
